# KATZLDA: KATZ measure for the lncRNA-disease association prediction

**DOI:** 10.1038/srep16840

**Published:** 2015-11-18

**Authors:** Xing Chen

**Affiliations:** 1 National Center for Mathematics and Interdisciplinary Sciences, Chinese Academy of Sciences, Beijing, 100190, China; 2 Academy of Mathematics and Systems Science, Chinese Academy of Sciences, Beijing, 100190, China

## Abstract

Accumulating experimental studies have demonstrated important associations between alterations and dysregulations of lncRNAs and the development and progression of various complex human diseases. Developing effective computational models to integrate vast amount of heterogeneous biological data for the identification of potential disease-lncRNA associations has become a hot topic in the fields of human complex diseases and lncRNAs, which could benefit lncRNA biomarker detection for disease diagnosis, treatment, and prevention. Considering the limitations in previous computational methods, the model of KATZ measure for LncRNA-Disease Association prediction (KATZLDA) was developed to uncover potential lncRNA-disease associations by integrating known lncRNA-disease associations, lncRNA expression profiles, lncRNA functional similarity, disease semantic similarity, and Gaussian interaction profile kernel similarity. KATZLDA could work for diseases without known related lncRNAs and lncRNAs without known associated diseases. KATZLDA obtained reliable AUCs of 7175, 0.7886, 0.7719 in the local and global leave-one-out cross validation and 5-fold cross validation, respectively, significantly improving previous classical methods. Furthermore, case studies of colon, gastric, and renal cancer were implemented and 60% of top 10 predictions have been confirmed by recent biological experiments. It is anticipated that KATZLDA could be an important resource with potential values for biomedical researches.

Sequence analysis indicates that more than 98% of the human genome doesn’t encode protein sequences and the proportion of non-coding sequence even significantly increases with the organism complexity^1^,^2^,^3^,^4^,^5^,^6^,^7^,^8^,^9^. Furthermore, growing evidences based on biological experiments have demonstrated that plenty of noncoding RNAs (ncRNAs) play critical roles in various fundamental and important biological processes^10^. According to transcript length, ncRNAs could be further categorized into small ncRNAs (such as miRNA, siRNA, piRNA) and long ncRNAs (lncRNA)^11^. LncRNAs are a large and important class of heterogeneous ncRNAs with a length more than 200 nucleotides^6^,^12^,^13^. Along with the rapid development of experimental technology and computational methods in the recent years, thousands of lncRNAs have been discovered in the eukaryotic organisms ranging from nematodes to humans^14^,^15^. Furthermore, the significant differences between lncRNAs and protein-coding genes have been revealed. For example, lncRNAs have lower cross-species conservation, much more tissue specificity, and relatively lower expression level^15^,^16^,^17^. Therefore, it is no surprise that people questioned the functionality of lncRNAs and considered them to be transcriptional noises in the past. However, increasing number of experimental studies in recent years have demonstrated many critical biological roles of lncRNAs in various important biological processes, such as cell differentiation, proliferation and apoptosis, transcriptional, post-transcriptional, and epigenetic regulation, cell cycle control, and so on^7^,^12^,^14^,^17^,^18^,^19^,^20^,^21^,^22^. Accumulating evidences have further demonstrated the important associations between lncRNAs and a broad range of complex human diseases^12^,^17^,^18^, such as breast cancer^23^, hepatocellular cancer^24^, prostate cancer^25^, colon cancer^26^, lung cancer^27^, leukemia^24^, cardiovascular diseases^28^, and neurodegenerative disorders^29^. Nowadays, lncRNAs have been further considered as potential biomarkers in human disease diagnosis, treatment, and prognosis and potential drug targets in drug discovery and disease treatment^30^.

Various lncRNA-related biological datasets have been generated and stored in well-known publicly available databases, such as NRED^31^, lncRNAdb^20^, NONCODE^32^, including the information of lncRNA sequence, expression, function, and so on. However, only a relatively limited number of lncRNAs have been linked with the development and progression of diseases. In the recent several years, researchers have begun to pay more attention to analyzing known lncRNA-disease associations, exploring their association mechanism, and identifying potential associations^21^,^33^,^34^. The benefits of lncRNA-disease association identification are manifold^21^,^34^. First, it could accelerate the understanding of human complex disease mechanism at the lncRNA level. Furthermore, more available disease-related lncRNAs could benefit disease biomarker detection and molecular tool design for disease diagnosis, treatment, prognosis, and prevention. Finally, it could effectively promote personalized medicine and human medical improvement. Computational models could quantify lncRNA-disease association probability and select the most probable associations for biological experimental validation. In this case, the number of candidate lncRNAs and the time and cost of experiment could be significantly decreased. Developing effective computational models and tools to predict potential disease-lncRNA associations has become a hot topic in the fields of human complex diseases and lncRNAs.

Some computational methods have been developed to predict novel disease-lncRNA associations, which could be classified into the following three categories. The first category is developing machine learning-based models to predict potential lncRNA-disease associations based on known disease-related lncRNAs. For example, based on the assumption that similar diseases are often associated with lncRNAs which have similar functions, Chen *et al.* developed the method of Laplacian Regularized Least Squares for LncRNA–Disease Association (LRLSLDA) in the semi-supervised learning framework to effectively identify potential disease–lncRNA associations by integrating known associations and lncRNA expression profiles^34^. In 2015, Chen *et al.* further developed two novel lncRNA functional similarity calculation models (LNCSIM) to calculate lncRNA functional similarity by measuring the semantic similarity between their associated disease groups^35^. Then, reliable performance improvement has been obtained based on cross validation and case studies about colorectal cancer and lung cancer when LRLSLDA was combined with LNCSIM. The second category is predicting novel lncRNA-disease associations based on random walk by integrating known lncRNA-disease association and similarity among lncRNAs or/and diseases. Most of these methods can’t be applied to new diseases without known associated lncRNAs and/or new lncRNAs without any known associated diseases or known miRNA interaction partners. For example, Sun *et al.* proposed a novel computational framework of RWRlncD to detect potential human lncRNA–disease associations by implementing random walk with restart on a lncRNA functional similarity network^36^. Recently, Zhou *et al.* constructed lncRNA–lncRNA crosstalk network by examining the significant co-occurrence of shared miRNA response elements on lncRNA transcripts, and proposed the model of RWRHLD to identify potential lncRNA–disease associations by implementing random walk with restart on the heterogeneous network^37^. The third category is constructing lncRNA-gene association network and obtaining potential lncRNA-disease associations based on known disease –related genes. These methods all strongly relied on disease related gene records. So these models can’t effectively predict potential related lncRNAs for the diseases with few or no related gene records. It has limited their wide applications. For example, based on hypergeometric distribution test, Liu *et al.*^38^ and Chen^39^ developed novel computational models to predict lncRNA-disease associations based on known disease-associated genes and miRNAs, respectively. In their studies, the relationship between lncRNAs and genes/miRNAs was calculated through expression profiles of lncRNAs and genes and known lncRNA-miRNA interactions, respectively. Li *et al.* presented a simple computational method to predict novel associations between lncRNAs and vascular disease based on genomic locations of vascular disease-related genes and candidate lncRNAs^40^. In addition, Yang *et al.* constructed a coding-non-coding gene-disease bipartite network based on known disease genes and disease-related ncRNAs and uncovered the hidden lncRNA-disease associations by implementing a global propagation algorithm on this network^41^.

In this study, I developed the model of KATZ measure for LncRNA-Disease Association prediction (KATZLDA) to predict potential lncRNA-disease associations by integrating known lncRNA-disease associations, lncRNA expression profiles, lncRNA functional similarity, disease semantic similarity, and Gaussian interaction profile kernel similarity for diseases and lncRNAs. Different from many previous computational models, KATZLDA could work for the lncRNAs without any known associated diseases and diseases without any known related lncRNAs. Leave-one-out cross validation (LOOCV) and 5-fold cross validation were implemented for KATZLDA based on known experimentally verified lncRNA-disease associations in the LncRNADisease database^21^. As a result, KATZLDA obtained reliable AUCs of 7175, 0.7886, 0.7719 in the local and global leave-one-out cross validation and 5-fold cross validation, respectively, significantly improving previous classical methods. Furthermore, case studies of colon cancer, gastric cancer, and renal cancer were implemented based on the prediction results of KATZLDA and 60% of top 10 predictions for these three important diseases have been confirmed by recent biological experiments. Both cross validation and case studies fully demonstrated the performance improvement over previous methods and potential value for disease-lncRNA association identification and lncRNA biomarker detection for human disease diagnosis, treatment, prognosis, and prevention.

## Results

### Model design

**Figure 1:**
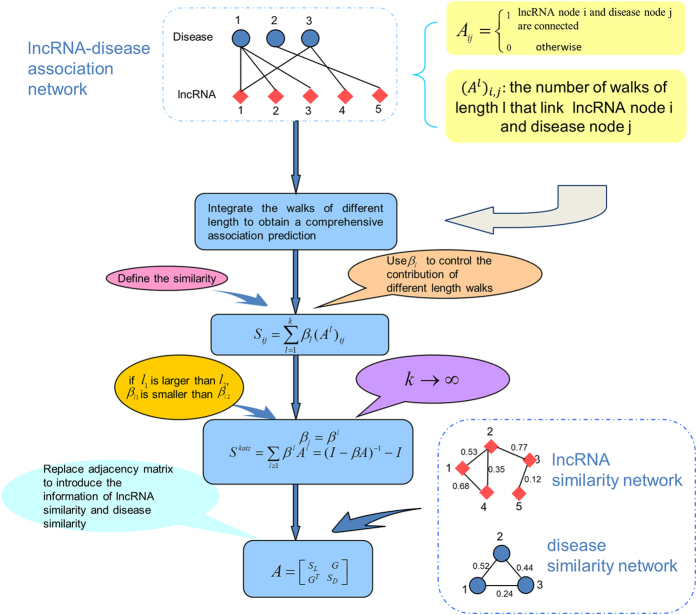
Flowchart of KATZLDA, demonstrating the basic ideas of adopting kATZ measure for lncRNA-disease association prediction.

KATZLDA was developed to predict potential disease-related lncRNAs by measuring the importance of candidate nodes relative to given seed nodes and identifying nodes similar to seed nodes (motivated by literature^42^,^43^, see Fig. 1). In the context of lncRNA-disease association prediction, KATZLDA computes the similarity scores between candidate lncRNAs and investigated diseases by integrating walks of different lengths between corresponding lncRNA and disease nodes (See the Method section for the detail of KATZLDA) in the heterogeneous network consisting of known disease-lncRNA association network, disease similarity network, and lncRNA similarity network. The novelty of KATZLDA could be largely attributed to the combination of the following several factors. Firstly, various types of biological datasets were integrated to implement the prediction, such as disease semantic similarity, lncRNA expression similarity, and lncRNA functional similarity (See the Method section for the detail of datasets used in this paper). New diseases (diseases without any known related lncRNAs) and lncRNAs (lncRNAs without any known associated diseases) are discovered each year. However, it is not clear whether newly discovered diseases would be correlated with some lncRNAs or uncorrelated with any lncRNAs. For these new diseases, KATZLDA could be used to quantify lncRNA-disease association probability and provide the potential lncRNA-disease pairs with higher association probability for biological experimental validation. If new disease is indeed related with some lncRNAs, KATZLDA could predict its potential related lncRNAs. The same conclusion is also true for newly discovered lncRNAs. Therefore, KATZLDA could work for both new diseases and lncRNAs. Finally, KATZLDA is a global method, which could reconstruct potential lncRNA-disease associations for all the diseases simultaneously. Therefore, KATZLDA represents an important and effective computational tool for biomedical research. Here, 293 distinct experimentally confirmed lncRNA–disease associations download from the LncRNADisease database were used as gold standard dataset in the cross validation for model evaluation and training dataset in the potential disease-lncRNA association prediction, respectively.

### Performance evaluation

Global and local LOOCV were implemented based on known experimentally verified lncRNA-disease associations in the lncRNADisease database to evaluate the performance of KATZLDA. When LOOCV was implemented, each known disease-lncRNA association was left out in turn as test sample and other known disease-lncRNA associations were regarded as training samples for model learning. The only difference between global and local LOOCV is the selection of candidate samples resulting from whether all the diseases were investigated simultaneously. For the global LOOCV, all the disease-lncRNA pairs without known relevance evidences would be considered as candidate samples. However, for the local LOOCV, attention is only paid to the disease in the test sample. Only all the lncRNAs without known associations with this disease would be regarded as candidate samples. How well the left-out test sample was ranked relative to candidate samples would be further evaluated. If the rank of test sample exceeds the given threshold, then the model was considered to implement a successful prediction. For the different thresholds, corresponding true positive rates (TPR, sensitivity) and false positive rates (FPR, 1-specificity) could be further obtained. Here, Sensitivity is the percentage of the test samples with the rank higher than the given threshold and specificity is the percentage of samples with the rank below this threshold. Therefore, Receiver-operating characteristics (ROC) curve could be drawn, which plots TPR versus FPR at different thresholds. Area under ROC curve (AUC) was calculated to evaluate the prediction performance of KATZLDA. AUC = 1 indicates perfect performance and AUC = 0.5 indicates random performance.

**Figure 2:**
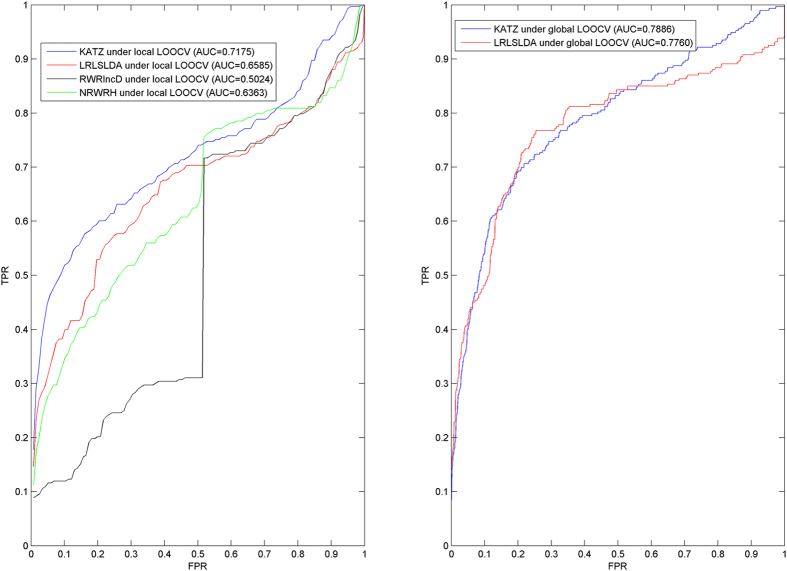
Performance comparisons between KATZLD and three the-state-of-art disease-lncRNA association prediction models (LRLSLDA, RWRlncD, and NRWRH) in terms of ROC curve and AUC based on LOOCV. As a result, KATZLDA achieved AUCs of 0.7886 and 0.7175 for the global and local LOOCV, respectively, which significantly improved all the previous classical models and effectively demonstrated its reliable predictive ability

KATZLD was compared with the following three the-state-of-art computational models in the framework of LOOCV: LRLSLDA^34^, RWRlncD^36^, and NRWRH^44^. LRLSLDA could reconstruct the missing associations for all the diseases simultaneously. Therefore, both global and local LOOCV could be implemented for LRLSLDA. However, global LOOCV can’t be implemented for RWRlncD and NRWRH because they only predict associated lncRNAs for the given disease. As a result, KATZLDA achieved AUCs of 0.7886 and 0.7175 for the global and local LOOCV, respectively (see Fig. 2). The performance of KATZLDA significantly improved all the previous classical models in the framework of both global and local LOOCV. LRLSLDA and RWRlncD can’t work for diseases without any known associated lncRNAs. Furthermore, RWRlncD and NRWRH can’t uncover the missing associations for all the diseases simultaneously. Therefore, except for significant improvement in the term of LOOCV, KATZLDA could effectively overcome these important limitations in the previous models.

Furthermore, 5-fold cross validation was implemented for KATZLDA. In the known lncRNA-disease association dataset, there were only about 1.75 known related lncRNAs for each disease and 2.48 known associated diseases for each lncRNA on average. Therefore, 5-fold cross validation was implemented based on all the known lncRNA-disease associations. All the known associations were randomly divided into 5-folds, i.e. 80% of the known associations were used as training samples for model learning, and the remaining 20% were used as test samples for model evaluation. All the disease-lncRNA pairs without known association evidences would be regarded as candidate samples.

As mentioned above, RWRlncD and NRWRH only could predict associated lncRNAs for the given disease and couldn’t infer all the missing associations for all the diseases simultaneously. Therefore, 5-fold cross validation couldn’t be implemented for these two computational models. Here, the comparison between KATZLDA and LRLSLDA based on 5-fold cross validation was implemented to further demonstrate the predictive ability of KATZLDA. To minimize the influence caused by sample division, the performance was evaluated under 100 different random divisions of known lncRNA-disease associations. ROC curves were drawn and AUCs were calculated for all the 100 experiments in the similar way to LOOCV, respectively. As a result, the mean and the standard deviation of AUCs for KATZLDA and LRLSLDA were 0.7719 +/– 0.0084 and 0.7295 +/– 0.0089, respectively. In conclusion, KATZLDA has demonstrated significant performance improvements over previous computational models in the evaluation framework of local LOOCV, global LOOCV, and 5-fold cross validation, respectively.

### Case studies

In order to further evaluate the predictive performance of KATZLDA, KATZLDA was applied to three kinds of important cancers for potential associated lncRNA prediction by regarding all the known disease-lncRNA associations as training samples for model learning. Prediction results were verified based on the recent updates in the LncRNADisease database and recently published experimental literatures. Validating prediction results in this framework for the model evaluation has been frequently adopted for previous computational models of disease related lncRNAs prediction^34^,^35^,^36^,^37^,^38^,^41^. Almost all the previous computational models reviewed in the Introduction section have been evaluated based on this framework. Furthermore, performance comparison between KATZLDA and LRLSLDA was implemented based on newly updated disease-lncRNA associations in the LncRNADisease database. All the updated associations for these three kinds of cancers have been checked and all the corresponding ranking results have been listed in Table 1.

Colon cancer is one of the most common malignant tumors worldwide and a great threat to public health^45^, even with the disease-specific mortality rate of nearly 33% in the developed world^46^. In China, the prevalence rate of colon cancer has increased dramatically in recent years due to the changes of human lifestyle^45^. Biological experiments have discovered some important association between the development and progression of colon cancer and mutations and dysregulations of lncRNAs^35^. KATZLDA was implemented to predict potential colon cancer-related lncRNAs. As a result, seven out of top ten potential related lncRNAs have been validated by the updates of lncRNADisease database^21^, MNDR database^47^ and recent biological experiments literature^48^. For example, the association between colon cancer and MALAT1, HOTAIR, UCA1, KCNQ10T1, and CRNDE (ranked 2nd, 4th, 6th, 7th, 9th in the prediction results, respectively) were validated by lncRNADisease database or MNDR database. Furthermore, according to The Cancer Network Galaxy (http://tcng.hgc.jp/index.html?t=gene&id=100048912), CDKN2B-AS1, 1st in the prediction results, has been included in many colon cancer-related networks constructed based on the expression data of primary colorectal cancers. Furthermore, real time PCR has indicated the expression level of PVT1 (3rd in the prediction results) in colon cancer tissues was higher than normal tissues and PVT1 was functionally correlated with the proliferation and invasion of colon cancer cells^46^,^48^. Therefore, it has been considered as a new oncogene in colon cancer tissues and an independent risk biomarker for overall survival of colon cancer patients^46^,^48^.

Gastric cancer is the second leading cause of cancer-related death and the fourth most common cancer worldwide^49^. Therefore, it is imperative to identify novel molecules for early diagnosis, prognosis, and treatment of gastric cancer. Accumulating evidences have demonstrated that lncRNAs have played critical roles in the de velopment and progression of gastric cancers^50^. KATZLDA was further implemented to identify lncRNAs potentially associated with gastric cancer. As a result, six out of top ten predicted lncRNAs have been validated by the updates of lncRNADisease database and recent biological experiment literatures^51^. H19, CDKN2B-AS1, MEG3, PVT1, and HOTAIR have been validated by lncRNADisease database, which was ranked 1st, 2nd, 3rd, 4th, and 7th in the prediction results, respectively. For example, both microarray and qRT-PCR have indicated that H19 was the most upregulated lncRNA among 135 differentially expressed lncRNAs in gastric cancer tissues relative to adjacent normal gastric mucosa^49^. In gastric cancer tissues, HOTAIR was also confirmed to exhibit abnormally high expression level relative to adjacent normal tissues^52^. The association between MALAT1 (5th in the prediction results) and gastric cancer has also been confirmed by experimental observations that MALAT1 was frequently upregulated in gastric cancer cell lines and could induce gastric cancer cell proliferation^51^.

Among the urinary system tumors, renal cancer has the third highest incidence, with more than 250,000 new cases diagnosed each year worldwide^53^. Nowadays, biological experiments have further discovered the associations between the development and progression of renal cancer and the mutations and dysregulations of some lncRNAs^53^. KATZLDA was applied to renal cancer for potentially related lncRNA prediction. As a result, five out of top ten predicted renal cancer-related lncRNAs have been validated by the update of lncRNADisease database and recent biological experiment literature reports. For example, H19, MEG3, PVT1, and MALAT1, ranked the 1st, 3rd, 4th, 6th in the prediction results, were validated by lncRNADisease database. Another confirmed lncRNA is UCA1, which was ranked the 8th in the prediction results. Biological experiments have shown that expression level of UCA1 in renal cancer tissue was significantly higher than normal tissues (http://www.cnki.com.cn/Article/CJFDTotal-ZLYD201507007.htm).

In addition, performance comparisons between KATZLDA and LRLSLDA were implemented based on the rankings of lncRNAs associated with colon, gastric, and renal cancer according to the updates of LncRNADisease database after gold-standard associations in this paper were downloaded (See Table 1). After getting rid of duplicate associations with different evidences and lncRNA-disease associations involved with lncRNAs which were not investigated in this paper, there were 19 distinct experimentally confirmed lncRNA–disease associations about these three important diseases. Observed results further indicated KATZLDA has more effective ability of inferring potential lncRNA-disease associations than LRLSLDA.

## Discussions

As valuable complements to experimental studies, computational models are in pressing need to effectively identify potential disease-related lncRNAs and lncRNA signature for disease diagnosis, therapeutic effect prediction, and treatment evaluation, considering the limitations of experimental methods and the generation of vast amount of biological datasets. In this article, KATZLDA was developed to predict potential lncRNA-disease associations on a large scale by integrating known lncRNA-disease associations, lncRNA expression profiles, lncRNA functional similarity, disease semantic similarity, and Gaussian interaction profile kernel similarity for diseases and lncRNAs to measure the importance of candidate lncRNAs relative to known disease-related lncRNAs. KATZLDA could be applied to new diseases and lncRNAs without any known associations. In order to validate reliable prediction performance of KATZLDA and demonstrate its advantage over previous classical models, local LOOCV, global LOOCV, and 5-fold cross validation were implemented based on known lncRNA-disease associations. Furthermore, case studies of colon cancer, gastric cancer, and renal cancer were implemented and 18 potential associations in the top 10 predictions for these three important diseases have been confirmed by recent experimental results. In the future, it is anticipated that KATZLDA could play important roles in potential lncRNA-disease association identification and disease biomarker detection.

Some limitations exist in the current version of KATZLDA. Firstly, although KATZLDA has significantly improved previous methods, its performance is still not very satisfactory, especially in the local LOOCV. Further data integration would benefit the improvement of predictive ability. For example, disease phenotypic similarity, known disease-genes/miRNAs associations, and various lncRNA-related interactions could be introduced into this model. Meanwhile, it is also very important to develop more effective similarity integration method. Furthermore, since Gaussian interaction profile kernel similarity and lncRNA functional similarity was calculated based on known lncRNA-disease associations, miRNA-disease associations, and lncRNA-miRNA interactions, KATZLDA may cause the bias to diseases with more known related lncRNAs and lncRNAs with more known associated diseases or/and more known miRNA interaction partners. Data integration would also benefit the decrease of the prediction bias. Thirdly, how to reasonably select nonnegative coefficients to differentiate the contribution from the different walks with different lengths is still not solved well. Finally, the new era of personalized medicine has dawned, so it is very important to design different models and different lncRNA biomarkers for different patients^54^,^55^,^56^.

## Methods

### LncRNA-disease associations

Known lncRNA-disease associations were downloaded from the LncRNADisease database in October, 2012^21^. After getting rid of duplicate associations with different evidences, there were 293 distinct experimentally confirmed lncRNA–disease associations about 118 lncRNAs and 167 diseases (see Supplementary Table 1). In order to use new associations added into this database after October, 2012 for the validation of potential lncRNA-disease associations predicted by KATZLDA, the latest version lncRNA-disease association dataset in the LncRNADisease database was not used as golden-standard dataset in this paper. Variable *nl* and *nd* represents the number of lncRNAs and diseases, respectively. Furthermore, matrix *A* is the adjacency matrix of lncRNA–disease association network. If lncRNA *l(i)* is related to the disease *d(j)*, *A(i,j)* is 1, otherwise 0.

### Disease semantic similarity

Furthermore, disease semantic similarity was calculated according to newly developed methods of constructing large-scale lncRNA functional similarity network^35^. Disease semantic similarity has been widely applied to identify disease-related ncRNAs and its effective performance has been fully demonstrated in plenty of previous studies^35^,^39^,^57^,^58^.

Disease semantic similarity would be calculated based on disease MeSH descriptors and their corresponding direct acyclic graphs (DAGs). Disease *A* can be described as *DAG(A)* = *(D(A),E(A))*, where *D(A)* is composed of the nodes of this disease itself and its ancestor diseases and *E(A)* consists of all the direct edges from parent nodes to child nodes. In the traditional disease semantic similarity calculation model^35^, the disease terms in the same layer would have the same contribution to the semantic value of disease *A*. However, considering the fact that two diseases in the same layer of *DAG(A)* may appear in the different numbers of disease DAGs, it is less accurate to assign the same contribution value to them. Based on the assumption that a more specific disease should have a greater contribution to the semantic value of disease *A*, the contribution of disease term t in *DAG(A)* was defined as follows:


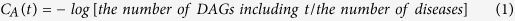


Therefore, the semantic value of disease *A* was obtained by summing all the contributions from ancestor diseases and disease *A* itself as follows.


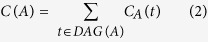


Furthermore, disease semantic similarity between disease *A* and *B* could be defined as follows by paying attention to the nodes shared by their corresponding disease DAGs:


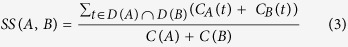


In this way, disease semantic similarity matrix *SS* could be constructed, where the entity *SS(i, j)* in row *i* column *j* is the disease semantic similarity between disease *d(i)* and *d(j)*.

### LncRNA expression similarity

Considering the fact that comprehensive lncRNA expression data has been unavailable till now and long intergenic non-coding RNA (lincRNA) occupies a large part of the whole lncRNA set, lincRNA expression profiles were downloaded from UCSC Genome Bioinformatics (http://genome.ucsc.edu/) in October, 2012, which included 21626 lincRNAs’ expression profiles across 22 human tissues or cell types (Supplementary Table 2). Then, lincRNA expression similarity was defined by calculating the Spearman correlation coefficient between the expression profiles of each lincRNA pair. Matrix *ES* represents the lncRNA expression similarity matrix, where *ES(i, j)* is the expression similarity between lncRNA *l(i)* and *l(j)* if they are both lincRNA, otherwise 0.

### LncRNA functional similarity

In the previous study, based on the assumption that lncRNAs with similar functions tend to interact with similar miRNAs and similar miNAs tend to be associated with similar diseases, the model of LFSCM was developed to calculate lncRNA functional similarity by integrating disease semantic similarity, miRNA-disease associations, and lncRNA-miRNA interactions^39^. Here, lncRNA functional similarity results in that study was introduced into the current study. Therefore, lncRNA functional similarity matrix *FS* could be obtained, where the entity *FS(i,j)* in row *i* column *j* is the functional similarity between lncRNA *l(i)* and *l(j)* according to the similarity calculation model of LFSCM.

### Gaussian interaction profile kernel similarity for diseases and lncRNAs

Based on the topology information of known lncRNA–disease association network and the assumption that similar diseases tend to show a similar interaction and non-interaction pattern with the lncRNAs, Gaussian interaction profile kernel similarity could be constructed for diseases^34^,^59^. Firstly, the interaction profile *IP (d(i))* of disease *d(i)* was defined as the *i*th column of the adjacency matrix *A*, which was a binary vector encoding the presence or absence of the known associations between disease *d(i)* and each lncRNA. Then, the Gaussian interaction profile kernel similarity between disease *d(i)* and *d(j)* was defined based on their interaction profiles as follows.


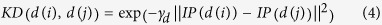



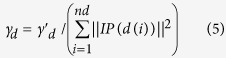


Here, the parameter γ_*d*_ controlled the kernel bandwidth, which was obtained by dividing a new bandwidth parameter 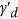 by the average number of associations with lncRNAs per disease. Therefore, disease Gaussian interaction profile kernel similarity matrix *KD* could be obtained, where the entity *KD(i,j)* is the Gaussian interaction profile kernel similarity between disease *d(i)* and *d(j)*.

LncRNA Gaussian interaction profile kernel similarity matrix *KL* can be constructed in the similar way:


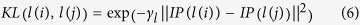


Here, *IP(l(i))* was the binary vector encoding the presence or absence of known associations between lncRNA *l(i)* and each disease. Parameter 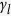 controlled the kernel bandwidth, which can be obtained by normalizing a new bandwidth parameter 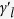.


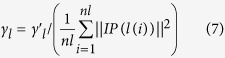


### Integrated similarity for diseases and lncRNAs

Based on the aforementioned disease semantic similarity, lncRNA expression similarity, lncRNA functional similarity, and Gaussian interaction profile kernel similarity, integrated disease similarity matrix *DS* and integrated lncRNA similarity matrix *LS* could be constructed as follows based on trivial combinatorial coefficients.


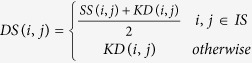



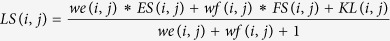


where *DS(i, j)* is the integrated similarity between disease *d(i)* and *d(j)*, *LS(i, j)* is the integrated similarity between lncRNA *l(i)* and *l(j)*, *IS* is the set of diseases with MeSH descriptors and tree numbers (disease semantic similarity could only be calculated for the diseases with both MeSH descriptors and tree numbers), *we(i, j)* is a binary variable indicating whether both of these two lncRNA are lincRNA, and *wf(i, j)* is a binary variable indicating whether both of these two lncRNAs have known functional similarity according to the similarity results calculated in the literature^39^. Here, trivial combinatorial coefficients were adopted according to previous similar studies, where robust performance has been demonstrated for various combinatorial coefficients and reliable predictive ability has been fully demonstrated based on cross validation and case studies^34^,^44^,^60^. Actually, further cross validation based on another independent dataset could be implemented to select these combinatorial coefficients.

### Katzlda

Inspired from the success of applying KATZ measure to link prediction in social networks and disease-gene association network^41^,^42^, the model of KATZLDA was developed to demonstrate its effectiveness for the identification of disease-lncRNA associations (See Fig. 1). KATZ is a graph-based computational method which transforms the problem of link prediction into a problem of calculating similarities between nodes in a heterogeneous network. The number of walks between nodes and walk lengths have been regarded as effective similarity metrics in the social network and biological network^41^,^42^,^61^,^62^. Therefore, in the context of lncRNA-disease association prediction, calculating similarities between the nodes of lncRNA and disease is further transformed into the problem of counting the number of walks that connect lncRNA node and disease node in the heterogeneous network. Furthermore, the number of walks and their lengths were integrated to decide the potential association probability of this lncRNA-disease pair. Here, heterogeneous network consists of disease similarity network constructed based on integrated disease similarity, lncRNA similarity network constructed based on integrated lncRNA similarity, and known disease-lncRNA association network constructed based on known associations downloaded from the lncRNADisease database.

KATZLDA only based on known disease-lncRNA association network is first introduced here. Therefore, the number of walks connecting lncRNA node *l(i)* and disease node *d(j)* in the known lncRNA-disease association network is calculated. It is easy to see 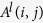 is exactly the number of walks of length *l* that link lncRNA node *l(i)* and disease node *d(j)*. In order to obtain a single similarity measure between these two nodes as the potential association probability between corresponding lncRNA and disease, different walks of different lengths are integrated. To differentiate the contribution of different walks of different lengths based on the assumption that walks with shorter lengths tend to contribute more to the similarity between two nodes, nonnegative coefficient sequence 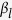 are introduced to dampen the contributions from longer walks by ensuring 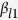 is smaller than 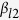 when 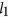 is larger than 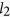. In this way, potential association probability between lncRNA *l(i)* and disease *d(j)* could be calculated based on the following formula.


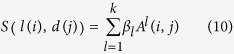


Here, I further let 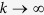, replace *β*_*l*_ by *β*^*l*^, and write above formula in the matrix form:


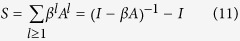


where the matrix *S* denotes the similarities between all the lncRNA-disease pairs. Above model only uses known disease-lncRNA associations. To make full use of the heterogeneous network constructed before, integrated disease similarity matrix *DS* and integrated lncRNA similarity matrix *LS* are further introduced into this computational model by replacing adjacency matrix *A* by the following form:


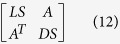


By integrating lncRNA and disease similarity, KATZLDA could be applied to new diseases and lncRNAs without any known associations.

## Additional Information

**How to cite this article**: Chen, X. KATZLDA: KATZ measure for the lncRNA-disease association prediction. *Sci. Rep.* **5**, 16840; doi: 10.1038/srep16840 (2015).

## Supplementary Material

Supplementary Information

Supplementary Table 1

Supplementary Table 2

## Figures and Tables

**Table 1:** Performance comparison between KATZLDA and LRLSLDA based on the rankings of newly discovered lncRNAs associated with Colon, Gastric, and Renal cancer, which were updated in LncRNADisease database.

Disease	lncRNA	KATZLDA	LRLSLDA
Colon cancer	MALAT1	2	3
Colon cancer	HOTAIR	4	15
Colon cancer	KCNQ1OT1	7	6
Colon cancer	CRNDE	9	32
Colon cancer	LSINCT5	85	115
Gastric cancer	H19	1	1
Gastric cancer	CDKN2B-AS1	2	2
Gastric cancer	MEG3	3	4
Gastric cancer	PVT1	4	3
Gastric cancer	HOTAIR	7	18
Gastric cancer	UCA1	11	16
Gastric cancer	LSINCT5	107	116
Gastric cancer	SPRY4-IT1	109	100
Renal cancer	H19	1	1
Renal cancer	MEG3	3	4
Renal cancer	PVT1	4	3
Renal cancer	MALAT1	6	9
Renal cancer	GAS5	62	63
Renal cancer	KCNQ1OT1	71	111
Average ranking		26.21	32.74

## References

[b1] Bertone P. *et al.* Global identification of human transcribed sequences with genome tiling arrays. Science 306, 2242–2246 (2004). 15539566 10.1126/science.1103388

[b2] Birney E. *et al.* Identification and analysis of functional elements in 1% of the human genome by the ENCODE pilot project. Nature 447, 799–816 (2007). 17571346 10.1038/nature05874 PMC2212820

[b3] Carninci P. *et al.* Genome-wide analysis of mammalian promoter architecture and evolution. Nat Genet 38, 626–635 (2006). 16645617 10.1038/ng1789

[b4] Claverie J. M. Fewer genes, more noncoding RNA. Science 309, 1529–1530 (2005). 16141064 10.1126/science.1116800

[b5] Core L. J., Waterfall J. J. & Lis J. T. Nascent RNA sequencing reveals widespread pausing and divergent initiation at human promoters. Science 322, 1845–1848 (2008). 19056941 10.1126/science.1162228 PMC2833333

[b6] Kapranov P. *et al.* RNA maps reveal new RNA classes and a possible function for pervasive transcription. Science 316, 1484–1488 (2007). 17510325 10.1126/science.1138341

[b7] Lander E. S. *et al.* Initial sequencing and analysis of the human genome. Nature 409, 860–921 (2001). 11237011 10.1038/35057062

[b8] Kapranov P., Willingham A. T. & Gingeras T. R. Genome-wide transcription and the implications for genomic organization. Nat Rev Genet 8, 413–423 (2007). 17486121 10.1038/nrg2083

[b9] Taft R. J., Pheasant M. & Mattick J. S. The relationship between non‐protein‐coding DNA and eukaryotic complexity. Bioessays 29, 288–299 (2007). 17295292 10.1002/bies.20544

[b10] Esteller M. Non-coding RNAs in human disease. Nat Rev Genet 12, 861–874 (2011). 22094949 10.1038/nrg3074

[b11] Hauptman N. & Glavač D. Long non-coding RNA in cancer. Int J Mol Sci 14, 4655–4669 (2013). 23443164 10.3390/ijms14034655 PMC3634483

[b12] Mercer T. R., Dinger M. E. & Mattick J. S. Long non-coding RNAs: insights into functions. Nat Rev Genet 10, 155–159 (2009). 19188922 10.1038/nrg2521

[b13] Guttman M., Russell P., Ingolia N. T., Weissman J. S. & Lander E. S. Ribosome profiling provides evidence that large noncoding RNAs do not encode proteins. Cell 154, 240–251 (2013). 23810193 10.1016/j.cell.2013.06.009 PMC3756563

[b14] Guttman M. *et al.* Chromatin signature reveals over a thousand highly conserved large non-coding RNAs in mammals. Nature 458, 223–227 (2009). 19182780 10.1038/nature07672 PMC2754849

[b15] Guttman M. *et al.* Ab initio reconstruction of cell type-specific transcriptomes in mouse reveals the conserved multi-exonic structure of lincRNAs. Nat Biotechnol 28, 503–510 (2010). 20436462 10.1038/nbt.1633 PMC2868100

[b16] Harrow J. *et al.* GENCODE: the reference human genome annotation for The ENCODE Project. Genome Res 22, 1760–1774 (2012). 22955987 10.1101/gr.135350.111 PMC3431492

[b17] Ponting C. P., Oliver P. L. & Reik W. Evolution and functions of long noncoding RNAs. Cell 136, 629–641 (2009). 19239885 10.1016/j.cell.2009.02.006

[b18] Wapinski O. & Chang H. Y. Long noncoding RNAs and human disease. Trends Cell Biol 21, 354–361 (2011). 21550244 10.1016/j.tcb.2011.04.001

[b19] Wilusz J. E., Sunwoo H. & Spector D. L. Long noncoding RNAs: functional surprises from the RNA world. Genes Dev 23, 1494–1504 (2009). 19571179 10.1101/gad.1800909 PMC3152381

[b20] Amaral P. P., Clark M. B., Gascoigne D. K., Dinger M. E. & Mattick J. S. lncRNAdb: a reference database for long noncoding RNAs. Nucleic Acids Res 39, D146–D151 (2011). 21112873 10.1093/nar/gkq1138 PMC3013714

[b21] Chen G. *et al.* LncRNADisease: a database for long-non-coding RNA-associated diseases. Nucleic Acids Res 41, D983–D986 (2013). 23175614 10.1093/nar/gks1099 PMC3531173

[b22] Khalil A. M. *et al.* Many human large intergenic noncoding RNAs associate with chromatin-modifying complexes and affect gene expression. Proc Natl Acad Sci U S A 106, 11667–11672 (2009). 19571010 10.1073/pnas.0904715106 PMC2704857

[b23] Gupta R. A. *et al.* Long non-coding RNA HOTAIR reprograms chromatin state to promote cancer metastasis. Nature 464, 1071–1076 (2010). 20393566 10.1038/nature08975 PMC3049919

[b24] Calin G. A. *et al.* Ultraconserved regions encoding ncRNAs are altered in human leukemias and carcinomas. Cancer Cell 12, 215–229 (2007). 17785203 10.1016/j.ccr.2007.07.027

[b25] de Kok J. B. *et al.* DD3PCA3, a very sensitive and specific marker to detect prostate tumors. Cancer Res 62, 2695–2698 (2002). 11980670

[b26] Pibouin L. *et al.* Cloning of the mRNA of overexpression in colon carcinoma-1: a sequence overexpressed in a subset of colon carcinomas. Cancer Genet Cytogenet 133, 55–60 (2002). 11890990 10.1016/s0165-4608(01)00634-3

[b27] Ji P. *et al.* MALAT-1, a novel noncoding RNA, and thymosin β4 predict metastasis and survival in early-stage non-small cell lung cancer. Oncogene 22, 8031–8041 (2003). 12970751 10.1038/sj.onc.1206928

[b28] Klattenhoff C. A. *et al.* Braveheart, a long noncoding RNA required for cardiovascular lineage commitment. Cell 152, 570–583 (2013). 23352431 10.1016/j.cell.2013.01.003 PMC3563769

[b29] Faghihi M. A. *et al.* Expression of a noncoding RNA is elevated in Alzheimer’s disease and drives rapid feed-forward regulation of β-secretase. Nat Med 14, 723–730 (2008). 18587408 10.1038/nm1784 PMC2826895

[b30] Spizzo R., Almeida M., Colombatti A. & Calin G. Long non-coding RNAs and cancer: a new frontier of translational research&quest? Oncogene 31, 4577–4587 (2012). 22266873 10.1038/onc.2011.621 PMC3433647

[b31] Dinger M. E. *et al.* NRED: a database of long noncoding RNA expression. Nucleic Acids Res 37, D122–D126 (2009). 18829717 10.1093/nar/gkn617 PMC2686506

[b32] Bu D. *et al.* NONCODE v3. 0: integrative annotation of long noncoding RNAs. Nucleic Acids Res 40, D210–D215 (2012). 22135294 10.1093/nar/gkr1175 PMC3245065

[b33] Yang G., Lu X. & Yuan L. LncRNA: A link between RNA and cancer. Biochim. Biophys. Acta. 1839, 1097–1109 (2014). 25159663 10.1016/j.bbagrm.2014.08.012

[b34] Chen X. & Yan G.-Y. Novel human lncRNA–disease association inference based on lncRNA expression profiles. Bioinformatics 29, 2617–2624 (2013). 24002109 10.1093/bioinformatics/btt426

[b35] Chen X. *et al.* Constructing lncRNA functional similarity network based on lncRNA-disease associations and disease semantic similarity. Sci Rep 5, 11338 (2015). 26061969 10.1038/srep11338 PMC4462156

[b36] Sun J. *et al.* Inferring novel lncRNA–disease associations based on a random walk model of a lncRNA functional similarity network. Mol Biosyst 10, 2074–2081 (2014). 24850297 10.1039/c3mb70608g

[b37] Zhou M. *et al.* Prioritizing candidate disease-related long non-coding RNAs by walking on the heterogeneous lncRNA and disease network. Mol Biosyst 11, 760–769 (2015). 25502053 10.1039/c4mb00511b

[b38] Liu M.-X., Chen X., Chen G., Cui Q.-H. & Yan G.-Y. A computational framework to infer human disease-associated long noncoding RNAs. PLoS One 9, e84408 (2014). 24392133 10.1371/journal.pone.0084408 PMC3879311

[b39] Chen X. Predicting lncRNA-disease associations and constructing lncRNA functional similarity network based on the information of miRNA. Sci Rep 5, 13186 (2015). 26278472 10.1038/srep13186 PMC4538606

[b40] Li J. *et al.* A bioinformatics method for predicting long noncoding RNAs associated with vascular disease. Sci China Life Sci 57, 852–857 (2014). 25104459 10.1007/s11427-014-4692-4

[b41] Yang X. *et al.* A network based method for analysis of lncRNA-disease associations and prediction of lncRNAs implicated in diseases. PLoS One 9, e87797 (2014). 24498199 10.1371/journal.pone.0087797 PMC3909255

[b42] Katz L. A new status index derived from sociometric analysis. Psychometrika 18, 39–43 (1953).

[b43] Singh-Blom U. M. *et al.* Prediction and validation of gene-disease associations using methods inspired by social network analyses. PLoS One 8, e58977 (2013). 23650495 10.1371/journal.pone.0058977 PMC3641094

[b44] Chen X., Liu M. X. & Yan G. Drug-target interaction prediction by random walk on the heterogeneous network. Mol BioSyst 8, 1970–1978 (2012). 22538619 10.1039/c2mb00002d

[b45] Xue Y. *et al.* Genome-wide analysis of long noncoding RNA signature in human colorectal cancer. Gene 556, 227–234 (2015). 25456707 10.1016/j.gene.2014.11.060

[b46] Han D. *et al.* Long noncoding RNAs: Novel players in colorectal cancer. Cancer Lett 361, 13–21 (2015). 25754818 10.1016/j.canlet.2015.03.002

[b47] Wang Y. *et al.* Mammalian ncRNA-disease repository: a global view of ncRNA-mediated disease network. Cell Death Dis 4, e765 (2013). 23928704 10.1038/cddis.2013.292 PMC3763453

[b48] Takahashi Y. *et al.* Amplification of PVT-1 is involved in poor prognosis via apoptosis inhibition in colorectal cancers. Br J Cancer 110, 164–171 (2014). 24196785 10.1038/bjc.2013.698 PMC3887297

[b49] Guo X., Xia J. & Deng K. Long non-coding RNAs: emerging players in gastric cancer. Tumour Biol 35, 10591–10600 (2014). 25173641 10.1007/s13277-014-2548-y

[b50] Zhao J. *et al.* Long non-coding RNAs in gastric cancer: versatile mechanisms and potential for clinical translation. Am J Cancer Res 5, 907–927 (2015). 26045977 PMC4449426

[b51] Wang J. *et al.* MALAT1 promotes cell proliferation in gastric cancer by recruiting SF2/ASF. Biomed Pharmacother 68, 557–564 (2014). 24857172 10.1016/j.biopha.2014.04.007

[b52] Hajjari M., Behmanesh M., Sadeghizadeh M. & Zeinoddini M. Up-regulation of HOTAIR long non-coding RNA in human gastric adenocarcinoma tissues. Med Oncol 30, 670 (2013). 23888369 10.1007/s12032-013-0670-0

[b53] Zhou S., Wang J. & Zhang Z. An emerging understanding of long noncoding RNAs in kidney cancer. J Cancer Res Clin Oncol 140, 1989–1995 (2014). 24816785 10.1007/s00432-014-1699-y PMC11824088

[b54] Wang E. *et al.* Predictive genomics: A cancer hallmark network framework for predicting tumor clinical phenotypes using genome sequencing data. Semin Cancer Biol 30, 4–12 (2015). 24747696 10.1016/j.semcancer.2014.04.002

[b55] Wang E. *et al.* Cancer systems biology in the genome sequencing era: Part 1, dissecting and modeling of tumor clones and their networks. Semin Cancer Biol 23, 279–285 (2013). 23791722 10.1016/j.semcancer.2013.06.002

[b56] Wang E. *et al.* Cancer systems biology in the genome sequencing era: Part 2, evolutionary dynamics of tumor clonal networks and drug resistance. Semin Cancer Biol 23, 286–292 (2013). 23792107 10.1016/j.semcancer.2013.06.001

[b57] Wang D., Wang J., Lu M., Song F. & Cui Q. Inferring the human microRNA functional similarity and functional network based on microRNA-associated diseases. Bioinformatics 26, 1644–1650 (2010). 20439255 10.1093/bioinformatics/btq241

[b58] Xuan P. *et al.* Prediction of microRNAs Associated with Human Diseases Based on Weighted k Most Similar Neighbors. PLoS One 8, e70204 (2013). 23950912 10.1371/journal.pone.0070204 PMC3738541

[b59] van Laarhoven T., Nabuurs S. B. & Marchiori E. Gaussian interaction profile kernels for predicting drug–target interaction. Bioinformatics 27, 3036–3043 (2011). 21893517 10.1093/bioinformatics/btr500

[b60] Chen X., Liu M. X., Cui Q. H. & Yan G. Y. Prediction of Disease-Related Interactions between MicroRNAs and Environmental Factors Based on a Semi-Supervised Classifier. PloS one 7, e43425 (2012). 22937049 10.1371/journal.pone.0043425 PMC3427386

[b61] Krauthammer M., Kaufmann C. A., Gilliam T. C. & Rzhetsky A. Molecular triangulation: bridging linkage and molecular-network information for identifying candidate genes in Alzheimer’s disease. Proc Natl Acad Sci USA 101, 15148–15153 (2004). 15471992 10.1073/pnas.0404315101 PMC523448

[b62] Radivojac P. *et al.* An integrated approach to inferring gene–disease associations in humans. Protein 72, 1030–1037 (2008). 10.1002/prot.21989 PMC2824611 18300252

