# Supplementary material for: KATZLDA: KATZ measure for the lncRNA-disease association prediction
Source: Sci Rep. 2015 Nov 18;5:16840. doi: 10.1038/srep16840 (PMC4649494; doi:10.1038/srep16840)
Supplement: Supplementary Information [file srep16840-s1.docx]

**KATZLDA: KATZ measure for the lncRNA-disease association prediction**

Xing Chen^1, 2,*^

^1^National Center for Mathematics and Interdisciplinary Sciences, Chinese Academy of Sciences, Beijing, 100190, China

^2^Academy of Mathematics and Systems Science,

Chinese Academy of Sciences, Beijing, 100190, China

*Corresponding authors

**Email**: [xingchen@amss.ac.cn](mailto:xingchen@amss.ac.cn)

**Supplementary Information**

**Supplementary Table 1.** Known lncRNA-disease association dataset was downloaded from the LncRNADisease database in October, 2012. After getting rid of duplicate associations with the different evidences, there are 293 distinct experimentally confirmed lncRNA–disease associations about 118 lncRNAs and 167 diseases

**Supplementary Table 2.** LincRNA expression profiles dataset was downloaded from UCSC Genome Bioinformatics (<http://genome.ucsc.edu/>) in October, 2012, which included 21626 lincRNAs’ expression profiles in 22 human tissues or cell types.
